# Supplementary material for: Causal Relationship between Aging and Anorexia Nervosa: A White-Matter-Microstructure-Mediated Mendelian Randomization Analysis
Source: Biomedicines. 2024 Aug 16;12(8):1874. doi: 10.3390/biomedicines12081874 (PMC11351342; doi:10.3390/biomedicines12081874)
Supplement: Supplementary file 1 [file biomedicines-12-01874-s001.zip › Supplementary Figure/Figure S4/Singe SNP plot of MR analysis for TL to ubm-b-2106.pdf]

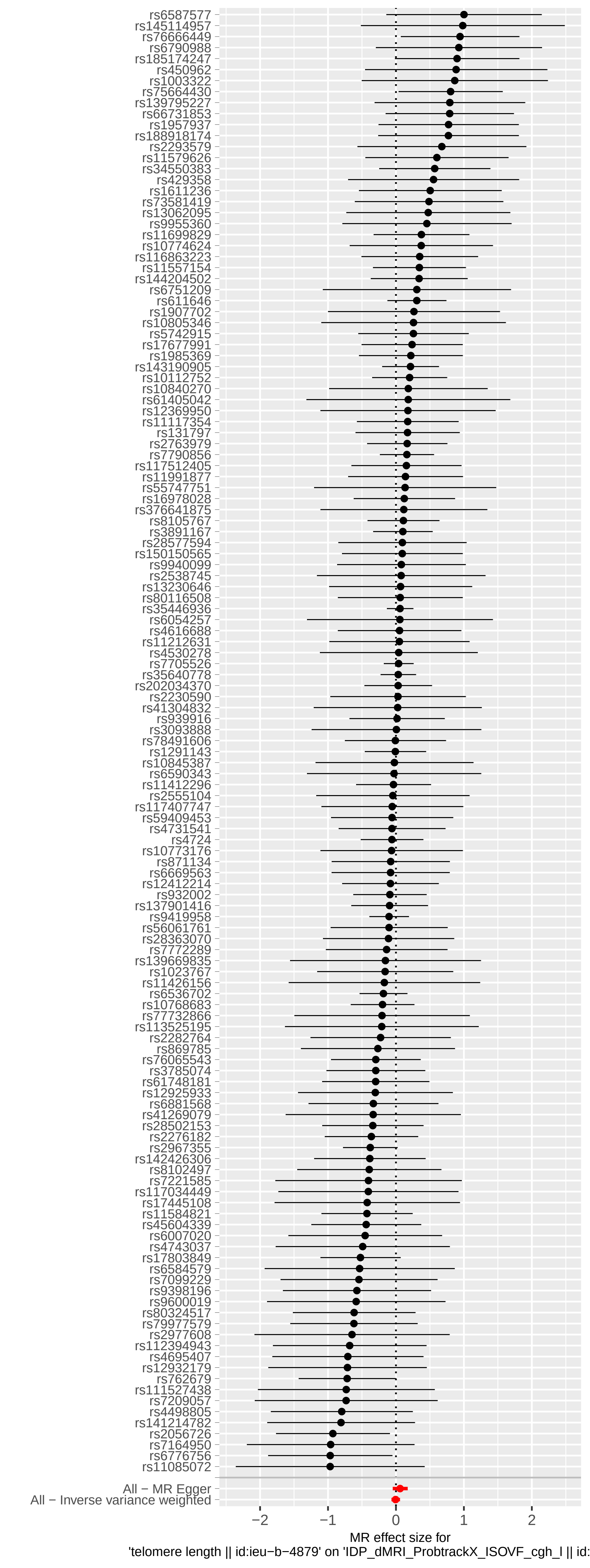
rs6587577
rs145114957
rs76666449
rs6790988
rs185174247
rs450962
rs1003322
rs75664430
rs139795227
rs66731853
rs1957937
rs188918174
rs2293579
rs11579626
rs34550383
rs429358
rs1611236
rs73581419
rs13062095
rs9955360
rs11699829
rs10774624
rs116863223
rs11557154
rs144204502
rs6751209
rs611646
rs1907702
rs10805346
rs5742915
rs17677991
rs1985369
rs143190905
rs10112752
rs10840270
rs61405042
rs12369950
rs11117354
rs131797
rs2763979
rs7790856
rs117512405
rs11991877
rs55747751
rs16978028
rs376641875
rs8105767
rs3891167
rs28577594
rs150150565
rs9940099
rs2538745
rs13230646
rs80116508
rs35446936
rs6054257
rs4616688
rs11212631
rs4530278
rs7705526
rs35640778
rs202034370
rs2230590
rs41304832
rs939916
rs3093888
rs78491606
rs1291143
rs10845387
rs6590343
rs11412296
rs2555104
rs117407747
rs59409453
rs4731541
rs4724
rs10773176
rs871134
rs6669563
rs12412214
rs932002
rs137901416
rs9419958
rs56061761
rs28363070
rs7772289
rs139669835
rs1023767
rs11426156
rs6536702
rs10768683
rs77732866
rs113525195
rs2282764
rs869785
rs76065543
rs3785074
rs61748181
rs12925933
rs6881568
rs41269079
rs28502153
rs2276182
rs2967355
rs142426306
rs8102497
rs7221585
rs117034449
rs17445108
rs11584821
rs45604339
rs6007020
rs4743037
rs17803849
rs6584579
rs7099229
rs9398196
rs9600019
rs80324517
rs79977579
rs2977608
rs112394943
rs4695407
rs12932179
rs762679
rs111527438
rs7209057
rs4498805
rs141214782
rs2056726
rs7164950
rs6776756
rs11085072
All − MR Egger
All − Inverse variance weighted
−2
−1
0
1
2
MR effect size for
'telomere length || id:ieu−b−4879' on 'IDP_dMRI_ProbtrackX_ISOVF_cgh_l || id:
